# Rate-Type Age-Dependent Constitutive Formulation of Concrete Loaded at an Early Age

**DOI:** 10.3390/ma12030514

**Published:** 2019-02-08

**Authors:** Seung-Gyu Kim, Yeong-Seong Park, Yong-Hak Lee

**Affiliations:** Depertment of Civil and Environmental Engineering, Konkuk University, 120 Neungdong-ro Gwangjin-gu, Seoul 05029, Korea; sgdandy@konkuk.ac.kr (S.-G.K.); parkys092@naver.com (Y.-S.P.)

**Keywords:** concrete, creep, shrinkage, time-varying elastic modulus, age-dependent constitutive model

## Abstract

A general formulation framework for an age-dependent constitutive equation of concrete is presented to account for the development of the elastic modulus at an early age. This is achieved by expanding the total stress vs. strain relation with respect to the time-varying elastic modulus. Two types of constitutive formulation frameworks are derived depending on whether (or not) the time-varying effect of the elastic modulus was taken into account in the linearized series expansion. The causes for the age-dependent deformations under sustained loads are defined in the formulation based on the two internal mechanisms of delayed elasticity and the ageing phenomenon. The ageing phenomenon is incorporated in a conventional delayed strain concept in terms of the variable elastic modulus with time. Four cases of age-dependent constitutive equations are formulated within the presented formulation framework by employing different types of creep models. The mechanical characteristics of the terms that comprise the various constitutive equations are examined and compared. Numerical application of the time-dependent test results of cylindrical specimens indicate that the creep formulation that considered the elastic modulus development showed a good agreement with the experimental result while the formulation that did not consider the elastic modulus development underestimated the result by 15%.

## 1. Introduction 

At present, with the advances in concrete and construction technologies, concrete formworks are removed at an early concrete age during the construction of concrete structures in order to reduce the work term. When stresses are introduced into concrete at an early age, the time-dependent displacement analysis of concrete structures should take into account the concrete characteristics during the early ages. Concrete properties during the early ages undergo significant development in terms of mechanical properties such as compressive and tensile strength, elastic modulus, creep, and shrinkage. Several major properties at the early ages including creep, strength, elastic modulus, degree of hydration, and crack formation were identified by intensive experimental works [1,2,3,4,5]. Among these properties, the elastic modulus develops rapidly at an early age, and the ratios of the elastic moduli at days 7 and 90 to that at day 28 are normally equal to 0.7 and 1.1, respectively [1,2,3,6]. Once the creep and shrinkage strains in a concrete structure are restrained, the time-varying value of elastic modulus as well as the mechanical strain due to a restraining effect is a significant factor in determining the internal stress state that persistently changes with time [7,8,9]. The analysis of concrete structures should account for the development of the elastic modulus at an early age as well as the persistent change in creep-causing internal stress. This paper presents a general formulation framework for an age-dependent constitutive equation of concrete to account for the ageing phenomenon of the elastic modulus development and the creep phenomenon with stress history in the creep model level as well as in the constitutive formulation level.

Several mechanisms are responsible for the development of creep, including the viscous flow in cement gels, seepage of water expulsion, delayed elasticity of the elastic deformations of cement gel and gel crystals, and breakage and reformation of bonds within the colloidal microstructures [6,10]. The complex internal mechanism of creep development is generally characterized as a “delayed” phenomenon that gradually progresses with time immediately after the development of the load-induced strain. Another recent aspect in the creep and shrinkage phenomena is a dissipative mechanism and the related plasticity due to the age-dependent deformation process, which is a typical problem in heterogeneous materials such as concrete [11,12]. However, this paper focuses on the development of age-dependent properties with time from a macroscopic perspective. The term “delayed” has a broader meaning than creep, and accounts for both the ageing and creep phenomena [10]. Ageing is caused by the chemical process of hydration, where the tricalcium silicate hydrate gel gradually fills the pores of hardened cement paste, resulting in the development of strength and elastic modulus. The effective modulus of Faber [13] is an early creep model that introduced a creep compliance function based on the concept of a “delayed” phenomenon, where all the time-dependent processes after loading were categorized as creep without distinguishing between creep and ageing. This paper defines the causes of the age-dependent deformations under sustained loads with the two internal mechanisms of delayed elasticity and the ageing phenomenon, where the ageing phenomenon is incorporated in a conventional delayed strain concept in terms of the variable elastic modulus with time.

The age-dependent constitutive law is normally formulated within a two-fold formulation framework of the material and mechanical aspects, whereby the age-dependent developments of creep, shrinkage, and elastic modulus, are formulated in the material aspect, while the age-dependent stress-strain relation is formulated in the mechanical aspect. The general forms of the creep models are expressed as a multiplication of the creep compliance function by the creep-inducing stress, where the compliance function represents the creep strain due to constant unit stress, which is referred to as a single creep curve because of its single functional form. ACI 209.2R–08 [14] introduced four creep prediction models, namely ACI 209R–92, B3 [15], GL 2000 [16], and CEB MC 90–99 [17], which were established based on numerous tests. Currently, these models are extensively utilized in creep analyses, and as a basis for further development [18,19,20,21,22,23,24,25]. Several methods have been presented to depict the development of creep strain under time history loading, including the effective modulus method (EMM) [13], rate of creep method (RCM) [26], and the ageing coefficient method (ACM) [10]. Recently, the parallel creep method (PCM) [27] was presented to account for the creep behaviors under a time-varying stress-history by combining the creep curve of RCM with that of EMM to account for the effects of age on creep upon loading. Three creep models, namely EMM, ACM, and PCM, are employed in this paper to define the creep terms in the constitutive formulation, where two types of creep functions including those with and without consideration of the elastic modulus development are accounted for in each creep model.

A constitutive formulation that features both the material and mechanical aspects was recently presented by Park and Lee [9], and it expanded the age-dependent total stress-strain relation using a first-order Taylor series expansion with respect to the three age-dependent material characteristics of creep, shrinkage, and elastic modulus. Based on this research work, this study presents a general formulation framework for the age-dependent constitutive equation to accommodate creep models derived in terms of the creep compliance function. Four age-dependent constitutive equations are explicitly formulated by the presented formulation framework with different types of creep models. The mechanical characteristics and the roles of the terms comprising the various constitutive equations are examined and compared. Creep tests of cylindrical concrete specimen subjected to stepwise loads, and the age-dependent behavior of an axially reinforced compressive member, are analyzed using the four constitutive equations examined herein, and the time-dependent behaviors at an early concrete age are addressed.

## 2. Delayed Strain Concept of the Creep Model

An early creep model of “delayed” phenomenon concept of creep is the effective modulus method [13] that introduced the creep compliance function to bring “delayed” concept into a mathematical form. In this method, all the time-dependent processes after loading are categorized into creep without distinguishing between creep and ageing. The delayed strain under single stress history $\sigma (t_{0})$ is expressed in terms of the creep compliance function $J(t,t_{0})$ and creep coefficient $\varphi (t,t_{0})$ as follows,

(1) $$\varepsilon (t)=\frac{1+\varphi (t,t_{0})}{E(t_{0})}\sigma (t_{0})+\varepsilon_{sh}(t)=J(t,t_{0})\sigma (t_{0})+\varepsilon_{sh}(t),$$

where $J(t,t_{0})=\left\{1/E(t_{0})\right\}+J^{\prime }(t,t_{0})$, $J^{\prime }(t,t_{0})$ is the creep function, $t_{0}$ is the time at loading ($t_{0}<t$). Denoting the immediate and elastic, creep and shrinkage strains at time t as $\varepsilon_{e}(t_{0})$ and $\varepsilon_{cr}(t)$, and $\varepsilon_{sh}(t)$, respectively, and assuming independence between creep and shrinkage [14,17], the total strain in Equation (1) can be expressed as the combination of these three components

(2) $$\varepsilon (t)=\varepsilon_{e}(t_{0})+\varepsilon_{cr}(t)+\varepsilon_{sh}(t),$$

Based on the relationship of Equation (2), creep strain can be calculated as

(3) $$\varepsilon_{cr}(t)=J^{\prime }(t,t_{0})\sigma (t_{0})=J(t,t_{0})\sigma (t_{0})-\frac{\sigma (t_{0})}{E(t_{0})},$$

It is observed from Equations (1) and (3) that the delayed concept of this creep model does not account for the ageing phenomenon in concrete because the value of the elastic modulus maintains a constant value for $E(t_{0})$ at the time of loading $t_{0}$, throughout the entire age-dependent process of concern. This approach may be acceptable in the case where concrete has an adequate time from casting before its loading, whereby the development of the elastic modulus becomes insignificant. However, this is not the case when the concrete is loaded at an early age, whereby the elastic modulus rapidly develops, and the amount of this development is not negligible. There exists a disagreement between the creep model in the material aspect and the constitutive model in the mechanical aspect when the creep model does not account for the time-dependent development of elastic strain and when the constitutive model accounts for the development of the elastic modulus. This disagreement could be critical when concrete is unloaded at time t such that the immediate strain recovery follows the elastic modulus E(t) at the same time t. For this reason, Equation (3) is modified to account for the ageing effect in the creep model

(4) $$\varepsilon_{cr}(t)=J^{\prime }(t,t_{0})\sigma (t_{0})+\left\{\frac{\sigma (t_{0})}{E(t_{0})}-\frac{\sigma (t_{0})}{E(t)}\right\}=J^{\prime \prime }(t,t_{0})\sigma (t_{0}),$$

**Figure 1:**
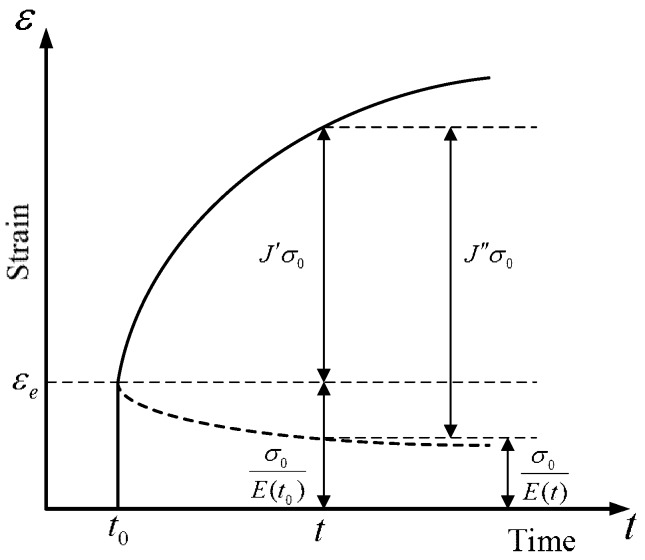
Schematic description of age-dependent strains.

The two terms in the parentheses of Equation (4) denote the ageing-induced creep strain owing to the development of the elastic modulus. Equation (4) recovers Equation (3) when the elastic modulus development with time is neglected, as is done in the case of the lack of ageing. Figure 1 illustrates the two cases of the delayed strain concepts regarding the elastic modulus, namely the cases with and without consideration of the ageing effect on the elastic modulus.

The two cases of creep definitions represented by Equations (3) and (4) are termed for convenience in this study as the $J^{\prime }$ and $J^{\prime \prime }$ concepts, respectively. In the case of multiple loads, the creep strain equation of Equation (4) is modified to multiple loadings at the ages of $t_{0}$, $t_{1}$, $t_{2}$, $t_{3}$, …, and $t_{n-1}$, as follows,

(5) $$\varepsilon_{cr}(t)=J^{\prime }(t,t_{0})\sigma (t)+\left\{\sum_{i=1}^{n}\frac{\sigma_{i-1}(t_{i-1})}{E(t_{i-1})}-\sum_{i=1}^{n}\frac{\sigma_{i-1}(t_{i-1})}{E(t)}\right\},$$

where $\sigma_{i-1}(t_{i-1})$ denotes the change of stress at time $t_{i-1}$ and has the same meaning as $\Delta \sigma (t_{i-1})$.

## 3. General Framework for Age-Dependent Constitutive Formulation

Creep and shrinkage lead to mechanical strain when their deformations are constrained. If an axial concrete member is subjected to shrinkage and creep whose deformations are restrained by reinforcement, the total stress-strain relation can be expressed as

(6) $$\sigma (t)=E(t)\;\varepsilon_{ms}(t)\;;\;\varepsilon_{ms}(t)=\varepsilon (t)-\varepsilon_{cs}(t),$$

where $\varepsilon_{ms}(t)$ and ε(t) denote the mechanical and member strains, respectively, at a current time t, $\varepsilon_{cs}(t)=\varepsilon_{cr}(t)+\varepsilon_{sh}(t)$, and the subscripts cr and sh denote creep and shrinkage, respectively. In the case where the ageing effect is neglected, such that the $J^{\prime }$ model is used as a creep model, the constant value of the elastic modulus $E(t_{0})$ at time $t_{0}$ replaces the time-varying elastic modulus E(t) of Equation (6) as

(7) $$\sigma (t)=E(t_{0})\left\{\varepsilon (t)-\varepsilon_{cs}(t)\right\},$$

The total stress-strain relation of Equation (6) is expressed by F(t) that is a function of creep strain $\varepsilon_{cr}(t)$, shrinkage strain $\varepsilon_{sh}(t)$, elastic modulus E(t), and the stress σ(t), as follows [9],

(8) $$F\{\sigma (t),\varepsilon_{cr}(t),\varepsilon_{sh}(t),E(t),t\}=\sigma (t)-E(t)\{\varepsilon (t)-\varepsilon_{cs}(t)\}=0,$$

When the elastic modulus, creep, and shrinkage, develop during the time change of $t-t_{0}$, the functional F(t) of Equation (8) violates the consistency condition, that is, F(t)≠0. To find the consistent state to be satisfied with the developments of three age-dependent variables, Equation (8) is expanded by a Taylor series expansion and is approximated by a first order expansion as [9]

(9) $$\dot{F}(t)={F(t=t_{n})+\frac{\partial F(t)}{\partial \sigma (t)}|}_{t=t_{n}}\dot{\sigma }(t)+{\frac{\partial F(t)}{\partial E(t)}|}_{t=t_{n}}{\dot{E}}_{c}(t)+{\frac{\partial F(t)}{\partial \varepsilon_{ms}(t)}|}_{t=t_{n}}{\dot{\varepsilon }}_{ms}(t)=0,$$

where ${\partial F_{c}(t)/\partial E(t)|}_{t_{n}}$$=\varepsilon_{ms}(t_{n})$, ${\partial F(t)/\partial \varepsilon_{ms}(t)|}_{t_{n}}$$=E(t_{n})$, ${\dot{\varepsilon }}_{ms}(t)=$$\dot{\varepsilon }(t)-{\dot{\varepsilon }}_{ns}(t)$, and $t_{n}$ is the reference time with respect to which the function is expanded. Assuming that the function is expanded at $t_{n}=t$, the linearized consistency condition of Equation (9) leads to a differential form of the age-dependent stress-strain relation as

(10) $$\dot{\sigma }(t)=E(t)\left\{\dot{\varepsilon }(t)-{\dot{\varepsilon }}_{cs}(t)\right\}+\dot{E}(t)\left\{\varepsilon (t)-\varepsilon_{cs}(t)\right\},$$

where $\dot{E}(t)$ presents the rate of elastic modulus at a reference time and ${\dot{\varepsilon }}_{cs}(t)={\dot{\varepsilon }}_{cr}(t)+{\dot{\varepsilon }}_{sh}(t)$. It is noted that the stress change in Equation (10) is caused by three sources of time-varying material properties: creep, shrinkage, and elastic modulus. If no change is assumed for the elastic modulus, as in the case of Equation (7), the differential age-dependent stress-strain relation of Equation (10) is simplified to

(11) $$\dot{\sigma }(t)=E(t_{o})\left\{\dot{\varepsilon }(t)-{\dot{\varepsilon }}_{cs}(t)\right\},$$

The rate-type age-dependent constitutive equation, formulated based on either Equation (10) or Equation (11), requires the mathematical creep model to be expressed in a rate form. Assuming that there is a creep function $J^{\prime }(t,t_{0})$ in Equation (3) that is dependent on the time-varying stress σ(t), a rate form of the age-dependent constitutive equation is obtained by substituting the rate form of the creep strain ${\dot{\varepsilon }}_{cr}(t)=$$\dot{J^{\prime }}(t,t_{0})\sigma (t)$$+J^{\prime }(t,t_{0})\dot{\sigma }(t)$ into Equation (10) as

(12) $$\begin{array}{ll} \dot{\sigma }(t)= & E_{ce}(t)\dot{\varepsilon }(t)-E_{ce}(t)\left\{{\dot{J}}^{\prime }(t,t_{0})\sigma (t)+{\dot{\varepsilon }}_{sh}(t)\right\}+E_{ce}(t)\frac{\dot{E}(t)}{E(t)}\varepsilon_{ms}(t)\\ & E_{ce}(t)=\frac{E(t)}{1+J^{\prime }(t,t_{0})E(t)} \end{array},$$

The constitutive equation for the case of an elastic modulus with a constant value can be obtained by replacing E(t) in Equation (12) with $E(t_{0})$ and by setting $\dot{E}(t)=0$. A rate form of the age-dependent constitutive equation based on the $J^{\prime \prime }$ model concept can be derived in a similar manner to that for Equation (12) as follows,

(13) $$\begin{array}{c} \dot{\sigma }(t)=E_{ce}(t)\dot{\varepsilon }(t)-E_{ce}(t)\left\{\varepsilon_{\alpha a}(t)+{\dot{\varepsilon }}_{sh}(t)\right\}+E_{ce}(t)\frac{\dot{E}(t)}{E(t)}\varepsilon_{ms}(t)\\ E_{ce}(t)=\frac{E(t)}{1+J^{\prime }(t,t_{0})E(t)},\;\varepsilon_{\alpha a}(t)=\left\{\dot{J^{\prime }}(t,t_{0})+\frac{\dot{E}(t)}{E^{2}(t)}\right\}\sigma (t) \end{array},$$

It is interesting to note that the effective modulus of Equation (13) takes a similar form as that of the age-adjusted effective modulus (AEMM) except for the ageing coefficient $\chi (t,t_{0})$ that is originally based on the delayed strain concept [10].

## 4. Age-Dependent Constitutive Formulations

Two cases of age-dependent constitutive equations were formulated by applying the $J^{\prime }$ and $J^{\prime \prime }$ concepts of Equations (3) and (4) into the formulation frameworks of Equations (10) and (11). Two creep models of ACM and PCM were employed in rate forms as the underlying creep models in the constitutive formulation. 

ACM [10] introduces an ageing coefficient $\chi (t,t_{0})$ into the earlier concept of delayed strain of the effective modulus method (EMM) of Faber [13]. Denoting the creep function as $J^{\prime }(t,t_{0})$ for a single constant load, the creep strain due to initial stress $\sigma (t_{0})$, and the time-varying stress Δσ(t) during the time period $t-t_{0}$, is expressed as

(14) $$\varepsilon_{cr}(t)=J^{\prime }(t,t_{0})\sigma (t_{0})+\chi (t,t_{0})J^{\prime }(t,t_{0})\Delta \sigma (t),$$

PCM was recently presented to account for the creep behaviors under a time-varying stress-history [27]. The creep model was formulated by combining the creep curve of the rate of the creep method (RCM) with that of EMM to account for the effects of age on creep upon loading. The resulting creep strain of PCM is bounded between the strain responses associated with RCM and EMM, and is expressed as

(15) $$\begin{array}{ll} {\dot{\varepsilon }}_{cr}(t)= & {\dot{\varepsilon }}_{\alpha }(t)+{\dot{J^{\prime }}}_{\alpha }(t)\dot{\sigma }(t)\;;{\dot{J^{\prime }}}_{\alpha }(t)=(1-\alpha_{n}){{\dot{J}}^{\prime }}_{0}(t)+\alpha_{n}{{\dot{J}}^{\prime }}_{n}(t)\\ & {\dot{\varepsilon }}_{\alpha }(t)=(1-\alpha_{n})\left\{{{\dot{J}}^{\prime }}_{0}(t)\sigma (t_{n-1})\right\}+\alpha_{n}\left\{\sum_{i=1}^{n}{{\dot{J}}^{\prime }}_{i-1}(t)\sigma_{i-1}(t_{i-1})\right\} \end{array},$$

where the ageing factor $\alpha_{n}=0.75$ may be chosen in the case where no information is available [27].

Three creep models with basic forms and the ACM and PCM were employed to define the term ${\dot{\varepsilon }}_{cr}(t)$ in Equations (10) and (11). Two types of creep functions, including those with and without consideration of the elastic modulus development (Equations (3) and (4)), respectively, were accounted for in each creep model to examine the effect of the development of the elastic modulus on the constitutive equation. The characteristics of the six cases of creep models are explained in Table 1. Table 2 summarizes the resulting six creep equations that correspond to the six creep models of Table 1. Creep equations of cases 1, 3, and 5, are based on the $J^{\prime }$ concept that does not consider the effect of the elastic modulus development on creep, while those for cases 2, 4, and 6, are based on the $J^{\prime \prime }$ concept that considers the effect of the elastic modulus development on creep.

Nine cases of age-dependent constitutive equations were formulated based on the two formulation frameworks defined by Equations (10) and (11). Specifically, the formulation frame of Equation (10) considers the age-dependent development of the elastic development in the series expansion, while the formulation frame of Equation (11) does not account for it. Six cases of creep functions listed in Table 2 are substituted with the rate expression of creep function ${\dot{\varepsilon }}_{cr}(t)$ defined in Equations (10) and (11). Table 3 summarizes the characteristics of the nine constitutive equations that specify the type of creep function and the formulation framework. Constitutive equations of cases 1 to 6 in Table 3 are derived based on Equation (10), and respectively employ the creep model cases 1 to 6 listed in Table 2. Constitutive equations based on Equation (11) are derived in cases 7, 8, and 9, whereby the creep model cases of 1, 3, and 5 have the basic forms of PCM and ACM, respectively, and are substituted with the creep function ${\dot{\varepsilon }}_{cr}(t)$ in Equation (11). The $J^{\prime }$ concept of the creep model that neglects the effect of the elastic modulus development was employed in cases 7, 8, and 9, to maintain consistency between the creep model and the constitutive formulation. Nine constitutive equations corresponding to Table 3 are summarized in Table 4.

In Table 4, the first term is the effective modulus that determines the age-dependent stress vs. strain relation, the second and third terms are the residual stresses induced by the applied loads and mechanical strain, respectively. The constitutive equation for each case is obtained by summing the three terms, and is termed in this study as the age-dependent incremental tangent modulus (AITM). Comparing the effective moduli indicates that they depend on the characteristic of the underlying creep model of the elastic modulus, and on the type of the creep function. The form of the effective modulus of case 1 is common among the nine effective moduli, and is the same as the expression of the delayed strain concept of Equation (1). This indicates that the Taylor series expansion-based formulation provides a mathematical background for the delayed strain concept. Cases 7, 8, and 9, do not account for the elastic modulus development with time in series expansion. In these cases, the third term does not appear in the constitutive equation, but it appears in cases 1 through 6, i.e., the cases that account for the elastic modulus development in the series expansions. The constitutive equation of case 9 was derived by applying the creep equation of ACM of Equation (14) to the presented formulation of Equation (11). The equivalence between the constitutive equation of case 9 and AAEM presented by Bazant [10] can be assessed by representing Equation (14) in a total strain form by introducing the elastic strain and the time-varying stress Δσ(t) during the time period $t-t_{0}$ into Equation (14), as follows

(16) $$\varepsilon (t)=\frac{\sigma (t_{0})+\Delta \sigma (t)}{E(t_{0})}+J^{\prime }(t,t_{0})\sigma (t_{0})+\frac{1}{E(t_{0})}\left\{\chi (t,t_{0})E(t_{0})J^{\prime }(t,t_{0})\right\}\Delta \sigma (t)+\varepsilon_{sh}(t),$$

The relation between the incremental stress Δσ(t) and strain Δε(t) can be expressed as

(17) $$\begin{array}{ll} \Delta \sigma (t)= & {E^{\prime }}_{ce}(t,t_{0})\Delta \varepsilon (t)-{E^{\prime }}_{ce}(t,t_{0})\left\{J^{\prime }(t,t_{0})\sigma (t_{0})+\varepsilon_{sh}(t)\right\}\\ & {E^{\prime }}_{ce}=\frac{E(t_{0})}{1+\chi (t,t_{0})E(t_{0})J^{\prime }(t,t_{0})} \end{array},$$

Observing the equivalence between the two constitutive equations of case 9 in Table 4 and the AAEM defined by Equation (17), it is identified that the presented formulation is a generalized formulation framework of a rate-type, age-dependent, constitutive equation. Cases 5 and 9 were respectively derived using the same creep model of ACM with the corresponding types of series expansions of Equations (10) and (11). In this sense, the constitutive formulation of case 5 improves the performance of the conventional formulation of case 9 by allowing the elastic modulus development at an early age. 

## 5. Numerical Applications and Observations

Constitutive equations presented in Table 4 are applied to the creep behaviors of unreinforced cylindrical concrete specimens and the reinforced concrete column. The effect of the development of the elastic modulus on creep was examined at a creep model level by applying the two principal concepts of creep definitions of Equations (3) and (4) to the time-dependent laboratory tests of unreinforced cylindrical specimens. The value of the ageing coefficient $\chi (t,t_{0})$ for ACM was considered to be equal to 0.8 [10]. The value of the ageing factor $\alpha_{n}(t)$ for PCM was considered to be equal to 0.75 [9]. Performances of cases 3 through 9 in Table 4 were investigated at a constitutive model level by predicting the time-dependent behaviors of the axially compressed reinforced column. In the latter case of analyses, two cases of continually increasing loads with time were applied in order to prevent a possible decrease of creep-causing stress because the interaction between concrete and axial reinforcement under compressive external load caused stress increments in concrete under tension.

### 5.1. Creep on Unreinforced Cylindrical Concrete Specimens

The effect of the elastic modulus development on creep was examined by applying Equations (3) and (4) to two series of time-dependent laboratory tests, namely, A and B [9]. Test specimens for tests A and B were cast with a diameter of 150 mm and a height of 300 mm, and the water-cement ratio of 54% and 57%, respectively. Two compressive axial loading cases were respectively considered for the test series A and B and corresponded to constant pressure and stepwise loads. In the case of constant pressure, 6 MPa and 5 MPa axial pressures were applied to tests A and B at the respective ages of 10 and 7 days from casting. In the case of stepwise loads, the axial pressures of 6, 8, 10, and 11 MPa, were applied at the ages of 10, 16, 43, and 65 days, for test series A, and the axial pressures of 5, 7, 9, and 11 MPa, were applied at the ages of 7, 15, 36, and 43 days, for test series B, respectively. The elastic modulus was measured at the ages when the stepwise loads were applied. Empirical equations for the elastic modulus development are presented in Table 5. Creep strains were computed by subtracting the immediate elastic and shrinkage strains from the total strain measurements. Two types of creep functions, namely $J^{\prime }$ and $J^{\prime \prime }$ expressed by Equations (3) and (4), were obtained from the constant load cases of test series A and B, and are presented in Table 5. 

**Figure 2:**
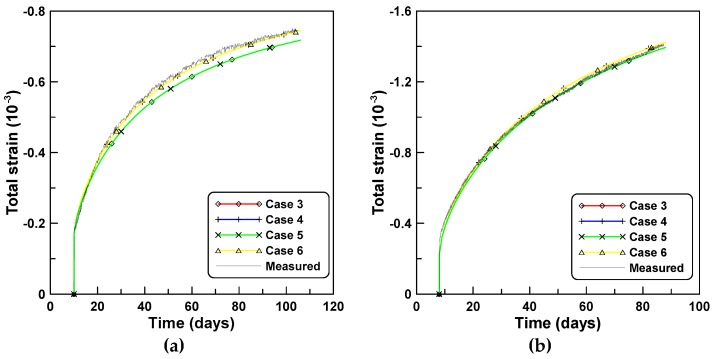
Total strains obtained by different creep models under constant loads: (**a**) test series A and (**b**) test series B.

**Figure 3:**
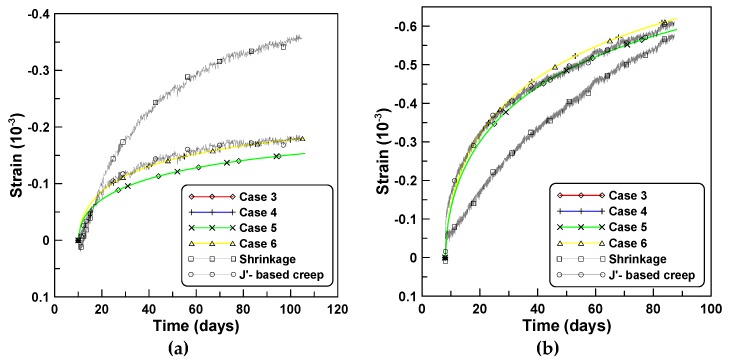
Shrinkage and creep strains elicited by different creep models under constant loads: (**a**) test series A and (**b**) test series B.

Figure 2a,b compares the total strains computed based on the two concepts of creep functions, $J^{\prime }$ and $J^{\prime \prime }$, with measurements from test series A and B, respectively. Cases 4 and 6 which employ $J^{\prime \prime }$ elicit a closer agreement compared to cases 3 and 5 for test series A. The differences between cases 3 and 5 and cases 4 and 6 can be calculated by estimating the differences between Equations (3) and (4) as $J^{\prime \prime }(t,t_{0})-J^{\prime }(t,t_{0})$$=1/E(t_{0})-1/E(t)$. Figure 3a,b compares the creep strains computed by different creep models where shrinkage and $J^{\prime }$-based creep are the measured shrinkage and creep strains, respectively, while the $J^{\prime }$-based creep was obtained by subtracting the immediate elastic and shrinkage strains from the total strain. 

**Figure 4:**
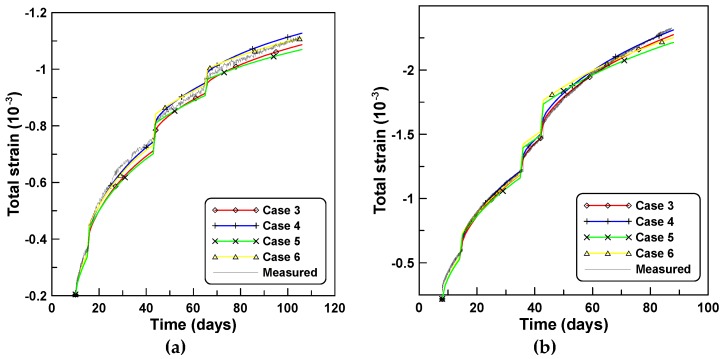
Total strains obtained by different creep models under stepwise loads: (**a**) test series A and (**b**) test series B.

**Figure 5:**
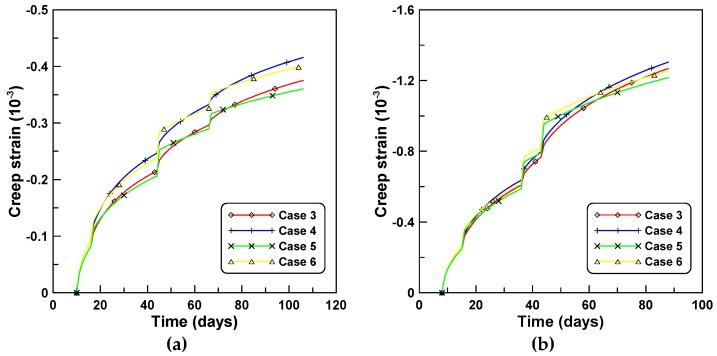
Creep strains obtained by different creep models under stepwise loads: (**a**) test series A and (**b**) test series B.

The four creep models of cases 3, 4, 5, and 6, listed in Table 2 are applied to the creep behaviors of unreinforced cylindrical specimen tests under stepwise loads for test series A and B. Figure 4a,b compare the total strains calculated by the four creep models for tests A and B, respectively. Differences between the two creep concepts of $J^{\prime }$ and $J^{\prime \prime }$ are observed in a similar manner to the constant load cases, whereby the test series A elicited a larger difference than test series B. Cases 3 and 5 employed the ACM and elicited a sudden increase of creep strain at the instant of load increase when the stepwise load was applied. This was because the ACM used the same creep function for the load increment $\Delta \sigma (t_{i})$ at time $t_{i}$ as the creep function for the first loading $\sigma (t_{0})$ at the initial time $t_{0}$. However, PCM yielded a smooth transition at the instants of load increase because PCM used the creep function for the load increment, according to the age of concrete at the instant of load increase. Figure 5 compares the creep strains calculated by the four creep models where the elastic modulus development had an increased influence on the magnitude of creep compared to the shape of the creep function.

**Figure 6:**
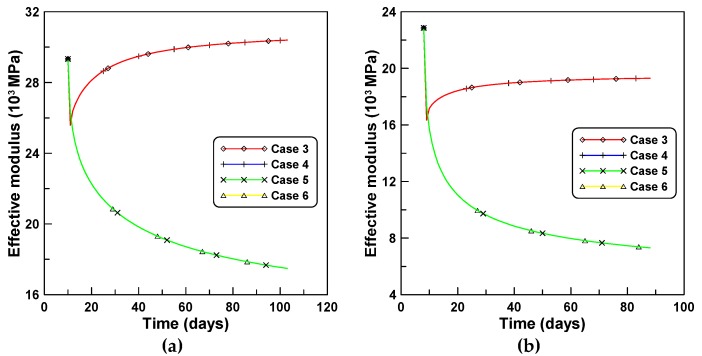
Variations of effective moduli of four creep models: (**a**) test series A and (**b**) test series B.

The variations of the effective moduli with time for the four creep models are compared in Figure 6a,b for tests A and B, respectively. The corresponding variations of the elastic moduli as a function of time for cases 3 and 5 are in close agreement with those for cases 4 and 6, respectively. This is because cases 3 and 4, and cases 5 and 6 were derived based on the same creep models of PCM and ACM, respectively, even though the creep concept of $J^{\prime }$ was used for cases 3 and 5, and the creep concept of $J^{\prime \prime }$ was used for cases 4 and 6. It is understood from this observation that the variation of the effective modulus with time is much more dependent on the type of the creep model rather than the type of the creep concept. Figure 6a,b indicate that age-dependent variations of effective moduli by PCM-based and ACM-based formulations are entirely different, whereby the results associated with the ACM case exponentially decay abruptly one day after the first loading, while the PCM case increases in a manner inverse to that of the ACM case. This is due to the fundamental difference between the PCM-based and ACM-based formulations. The former case is a rate-type formulation used to derive the tangent relation (modulus) between the incremental stress and strain changes within a small time increment, while the latter case is not a real rate-type formulation but rather a semi rate-type formulation, considering the relatively long time interval compared to the former case. 

### 5.2. Creep on Axially Reinforced Concrete Column

**Figure 7:**
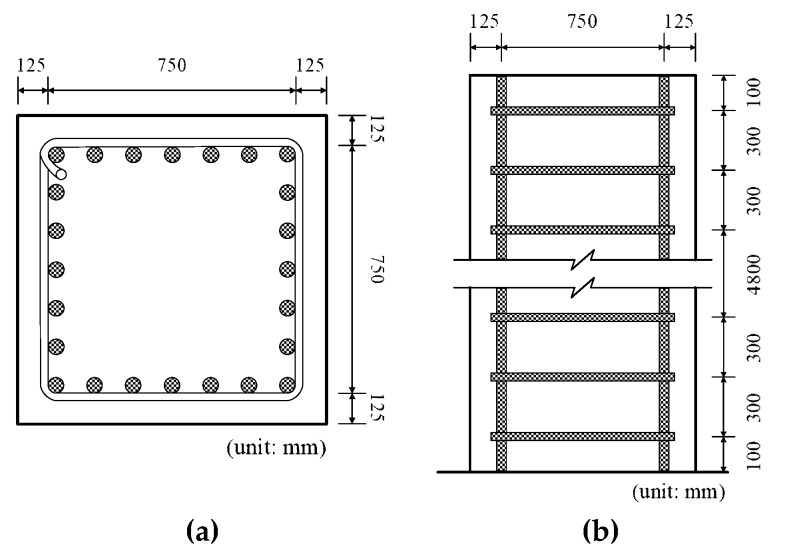
Dimensions of RC column: (**a**) cross-sectional dimension and (**b**) longitudinal dimension.

**Figure 8:**
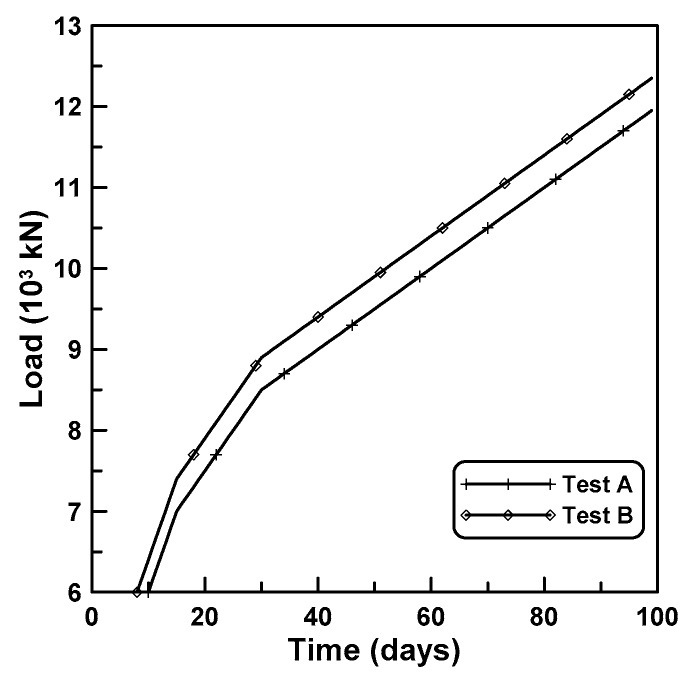
Two time-dependent load histories.

The time-dependent behaviors of a reinforced concrete column are analyzed according to the six cases of the constitutive formulations of cases 3, 4, 5, 6, 8, and 9, as listed in Table 4. A rectangular cross-section of the reinforced concrete (RC) column with a cross-sectional dimension of 1000 mm × 1000 mm is shown in Figure 7a where twenty-five axial reinforcements of D25 (nominal diameter: 25.4 mm) were placed with a center-to-center distance of 125 mm. Tied bars of D13 were placed with a center-to-center distance of 300 mm along the column height of 6 m (Figure 7b). Two cases of time-dependent analyses of A and B are considered with different age-dependent concrete properties and time-dependent load histories. Age-dependent concrete properties of tests A and B in Table 5 are employed to the analyses of cases A and B, respectively. Figure 8 shows the two cases of time-dependent load histories considered in the analyses of A and B. In case A, the initial load of 6000 kN was applied at the age of 10 days after casting. Additional loads of 1000 kN, 2250 kN, and 7000 kN, were linearly applied at the ages of 5 days, 15 days, and 70 days, respectively. In case B, the initial load of 6000 kN was applied at the age of 7 days after casting. Additional loads of 1600 kN, 2250 kN, and 7000 kN, were applied at the ages of 8 days, 15 days, and 70 days, respectively. The time-dependent behaviors were analyzed by using time-dependent finite element analysis because of the incremental analysis with time and the interaction between the concrete and reinforcements due to the restraining effect of reinforcement on age-dependent deformations. Finite beam element formulation was conducted to encompass the time-dependent flexural behavior though the current RC column behavior, which can be fully depicted by one-dimensional axial formulation. For this purpose, the concrete section and reinforcements were approximated by a conventional two-node beam element with six degrees of freedom per node and a two-node bar element with two degrees of freedom per node. Time-dependent finite element equilibrium equations were derived by using the conventional approach of the theorem of virtual work, where the presented constitutive equations were inserted into the virtual work formulation to define the age-dependent stress vs. strain relation. The equilibrium equations were linked with in-housing computer code of MIDAS [28].

**Figure 9:**
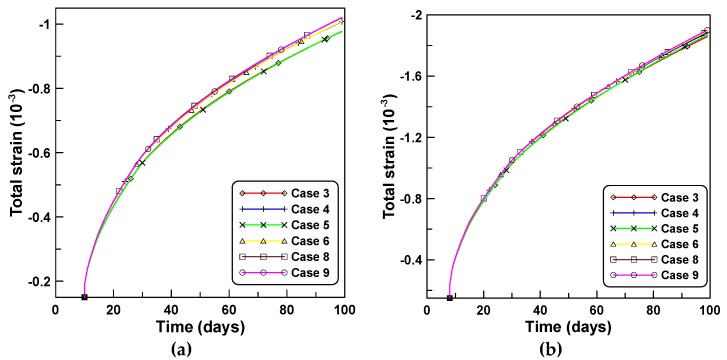
Total strains according to six constitutive formulations: (**a**) analysis of case A and (**b**) analysis of case B.

Figure 9a,b compares the total strains predicted by the six constitutive formulations of cases 3, 4, 5, 6, 8, and 9, for the analyses of series A and B, respectively. The total strain of case A is less than that of case B because of the diminished shrinkage strain owing to the lower W/C ratio in case A. It is similar to the total strains of unreinforced cylindrical specimen tests A and B shown in Figure 2. This is because the age-dependent material properties are used for the predictions of time-dependent behaviors of reinforced concrete column as well as the unreinforced specimen, as listed in Table 5.

**Figure 10:**
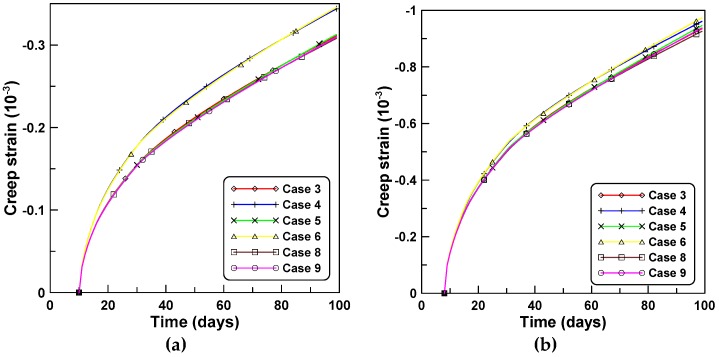
Creep strains by six constitutive formulations: (**a**) analysis of case A and (**b**) analysis of case B.

The effects of the time-varying creep concepts $J^{\prime }$ and $J^{\prime \prime }$, and the time-varying elastic modulus E(t) on the age-dependent behaviors are investigated by comparing the total strains computed for case A shown in Figure 9a. Close agreements are observed for the total strains of cases 3 and 5 and cases 4 and 6 that correspond to the comparison of the cases of the constitutive equations formulated with the same creep concepts $J^{\prime }$ and $J^{\prime \prime }$. Conversely, noticeable differences are observed for cases 3 and 4 and cases 5 and 6 that denote the cases used to compare the constitutive equations formulated based on the two creep concepts $J^{\prime }$ and $J^{\prime \prime }$, respectively. Close agreement in the total strains is also observed in cases 8 and 9 that were derived with the use of PCM-based and ACM-based creep models, respectively, based on the same creep concept of $J^{\prime }$. The difference in the total strains of cases 3 and 8 and cases 5 and 9 indicate the effects of time-varying elastic modulus E(t) on the age-dependent behaviors. Cases 3 and 5 that considered the variations of the elastic modulus with time predict smaller total strains compared to cases 8 and 9. It is also observed that the total strains of cases 4 and 6 are larger than those of cases 3 and 5. Figure 10a,b compare creep strains based on six constitutive equations, and correspond to the total strains of Figure 9, whereby creep strains are obtained by subtracting shrinkage and immediate elastic strain from the total strain. Creep strains based on cases 4 and 6 are larger than those for cases 3 and 5. The latter express a similar tendency to the tendency of the total strain case of Figure 9, whereby the total strains of cases 4 and 6 are larger than those of cases 3 and 5. 

**Figure 11:**
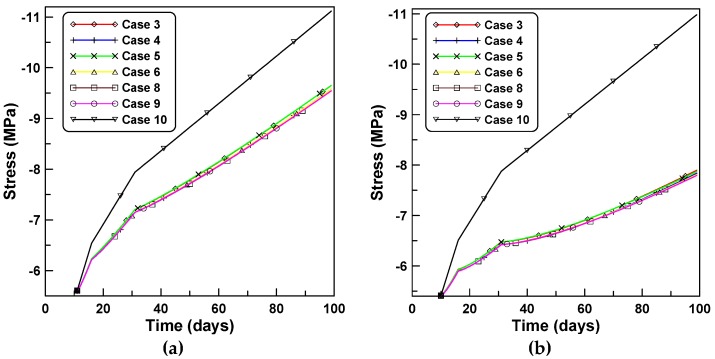
Concrete stresses based on six constitutive formulations: (**a**) analysis of case A and (**b**) analysis of case B.

**Figure 12:**
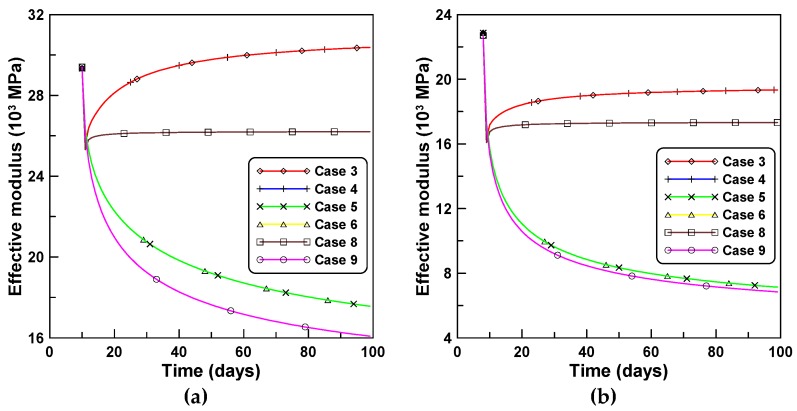
Effective moduli of six constitutive formulations: (**a**) analysis of case A and (**b**) analysis of case B.

Time-independent analyses were implemented for both analyses A and B to identify the age-dependent effect of concrete on the time-dependent RC column behavior. Accordingly, the concrete stress is plotted in case 10 and compared in Figure 11a,b with concrete stresses elicited by the cases 3, 4, 5, 6, 8, and 9. The concrete stress in the case of time-independent analysis is much larger than the concrete stresses elicited according to the six cases of the constitutive equations listed herein because the restraining effect of reinforcement on the lack of mechanical strain owing to shrinkage and creep induces mechanical stress in the concrete. Figure 12a,b compares the effective moduli of six constitutive formulations for cases A and B, respectively. The variations of the effective moduli with time for cases 3, 4, 5, and 6, show close agreements and similar patterns to those of unreinforced concrete specimens in Figure 6. 

## 6. Conclusions

An integrated constitutive formulation was presented to account for the development of elastic modulus at an early concrete age. Two types of constitutive formulation frameworks were derived depending on whether (or not) the age-dependent development of elastic modulus was taken into account in the formulation. Six different constitutive equations were formulated depending on the type of the underlying creep strain concept and the constitutive formulation framework, and they were applied to the age-dependent behaviors of unreinforced cylindrical concrete specimen tests and reinforced concrete column. The following conclusions were drawn:

1. Creep was divided into a delayed part of elastic deformation and an ageing part due to time-dependent chemical processes. This enabled incorporation of the ageing phenomenon in the conventional delayed strain concept by defining the ageing phenomenon in terms of the development of the elastic modulus. Furthermore, it ensured consistency in the two-fold formulation by allowing the use of the same time-varying elastic modulus in both the creep and constitutive formulation levels.
2. Two types of creep concepts—with and without consideration of the development of the elastic modulus—were applied to time-dependent tests of unreinforced cylindrical specimens. The creep concept that considered the development of elastic modulus showed a good agreement with the experimental result while the concept that did not consider the development of elastic modulus underestimated it by 15%. This observation indicates that the elastic modulus development needs to be considered in the creep model.
3. The presented formulation framework was used to derive six constitutive equations depending on the type of the creep concept and the condition based on which the elastic modulus development was considered as an expandable variable in a Taylor series expansion. The formulation process manifested an advantage of precisely figuring out the characters of the constitutive equation.
4. The comparison between the presented formulation framework and the constitutive equation of AAEM derived based on the ageing creep method verified the equivalence between the two constitutive models. This showed that the presented formulation is a generalized formulation formwork of a rate-type, age-dependent, constitutive equation, and provides a mathematical background for the conventional delayed strain concept in defining creep strain.
5. Numerical applications of the six constitutive equations to the RC column structure showed 4% difference in the time-dependent behavior of the particular RC column between the constitutive formulation cases with and without consideration of the development of elastic strain. This showed that the time-dependent behavior of the reinforced concrete structure was significantly dependent on the development of the elastic modulus.

## Figures and Tables

**Table 1:** Six cases of creep models.

Cases	Creep Models		
	Type of Creep Concept		Type of Creep Model
	$J^{\prime }$-Based	$J^{\prime \prime }$-Based	
Case 1	O	-	Basic form
Case 2	-	O	Basic form
Case 3	O	-	PCM
Case 4	-	O	PCM
Case 5	O	-	ACM
Case 6	-	O	ACM

**Table 2:** Creep model equations corresponding to the creep models.

Case (Creep Strain)	Creep Strain Rate $\left({\dot{\varepsilon }}_{cr}(t)\right)$	Total Creep Strain $\left(\varepsilon_{cr}(t)\right)$
Case 1	${\dot{J}}^{\prime }(t)\sigma (t)+J(t)\dot{\sigma }(t)$	$J^{\prime }(t)\sigma (t)$
Case 2	$\left\{\dot{J^{\prime }}(t)+\frac{\dot{E}(t)}{E^{2}(t)}\right\}\sigma (t)+J^{\prime }(t)\dot{\sigma }(t)$	$J^{\prime }(t)\sigma (t)+\left\{\sum_{i=1}^{n}\frac{\sigma_{i-1}(t_{i-1})}{E(t_{i-1})}-\sum_{i=1}^{n}\frac{\sigma_{i-1}(t_{i-1})}{E(t)}\right\}$
Case 3	${\dot{\varepsilon }}_{\alpha }(t)+{\dot{J^{\prime }}}_{\alpha }(t)\dot{\sigma }(t)$	$\int_{0}^{t}{\dot{\varepsilon }}_{cr}(t)dt$
Case 4	${\dot{\varepsilon }}_{\alpha }(t)+{\dot{J^{\prime }}}_{\alpha }(t)\dot{\sigma }(t)+\frac{\dot{E}(t)}{E^{2}(t)}\sigma (t)$	$\int_{0}^{t}{\dot{\varepsilon }}_{cr}(t)+\left\{\sum_{i=1}^{n}\frac{\sigma_{i-1}(t_{i-1})}{E(t_{i-1})}-\sum_{i=1}^{n}\frac{\sigma_{i-1}(t_{i-1})}{E(t)}\right\}$
Case 5	$\dot{J^{\prime }}(t)\sigma (t_{0})+\chi (t)J^{\prime }(t)\dot{\sigma }(t)$	$J^{\prime }(t)\sigma (t_{0})+\chi (t)J^{\prime }(t)\Delta \sigma (t)$
Case 6	$\dot{J^{\prime }}(t)\sigma (t_{0})+\chi (t)J^{\prime }(t)\dot{\sigma }(t)+\frac{\dot{E}(t)}{E^{2}(t)}\sigma (t)$	$\begin{array}{l} J^{\prime }(t)\sigma (t_{0})+\chi (t)J^{\prime }(t)\Delta \sigma (t)\\ +\left\{\sum_{i=1}^{n}\frac{\sigma_{i-1}(t_{i-1})}{E(t_{i-1})}-\sum_{i=1}^{n}\frac{\sigma_{i-1}(t_{i-1})}{E(t)}\right\} \end{array}$

(Notations: $\sigma (t_{0})$: load applied at time $t_{0}$, $\varepsilon_{cr}(t)$: total creep strain from $t_{0}$ to t).

**Table 3:** Characteristics of the nine constitutive equations.

Cases	Types of Formulation (Age-Dependent Stress-Strain Law)		
	Creep Model Case	Series Expansion with Respect to Elastic Modulus	
		Considered	Neglected
Case 1	Case 1	O	-
Case 2	Case 2	O	-
Case 3	Case 3	O	-
Case 4	Case 4	O	-
Case 5	Case 5	O	-
Case 6	Case 6	O	-
Case 7	Case 1	-	O
Case 8	Case 3	-	O
Case 9	Case 5	-	O

**Table 4:** Nine constitutive equations.

Cases	Time-Delay Modulus $\left(E_{ce}(t)\right)$	Residual Stress due to Applied Loads	Residual Stress due to Mechanical Strain
Case 1	$\frac{E(t)}{1+E(t)J^{\prime }(t)}$	$-E_{ce}(t)\left\{\dot{J^{\prime }}(t)\sigma (t)+{\dot{\varepsilon }}_{sh}(t)\right\}$	$E_{ce}(t)\frac{\dot{E}(t)}{E(t)}\varepsilon_{ms}(t)$
Case 2	$\frac{E(t)}{1+E(t)J^{\prime }(t)}$	$-E_{ce}(t)\left\{\left(\dot{J^{\prime }}(t)+\frac{\dot{E}(t)}{E^{2}(t)}\right)\sigma (t)+{\dot{\varepsilon }}_{sh}(t)\right\}$	$E_{ce}(t)\frac{\dot{E}(t)}{E(t)}\varepsilon_{ms}(t)$
Case 3	$\frac{E(t)}{1+E(t){\dot{J^{\prime }}}_{\alpha }(t)}$	$-E_{ce}(t)\left\{{\dot{\varepsilon }}_{\alpha }(t)+{\dot{\varepsilon }}_{sh}(t)\right\}$	$E_{ce}(t)\frac{\dot{E}(t)}{E(t)}\varepsilon_{ms}(t)$
Case 4	$\frac{E(t)}{1+E(t){\dot{J^{\prime }}}_{\alpha }(t)}$	$-E_{ce}(t)\left\{{\dot{\varepsilon }}_{\alpha }(t)+\frac{\dot{E}(t)}{E^{2}(t)}\sigma (t)+{\dot{\varepsilon }}_{sh}(t)\right\}$	$E_{ce}(t)\frac{\dot{E}(t)}{E(t)}\varepsilon_{ms}(t)$
Case 5	$\frac{E(t)}{1+\chi (t)E(t)J^{\prime }(t)}$	$-E_{ce}(t)\left\{\dot{J^{\prime }}(t)\sigma (t_{0})+{\dot{\varepsilon }}_{sh}(t)\right\}$	$E_{ce}(t)\frac{\dot{E}(t)}{E(t_{0})}\varepsilon_{ms}(t)$
Case 6	$\frac{E(t)}{1+\chi (t)E(t)J^{\prime }(t)}$	$-E_{ce}(t)\left\{\dot{J^{\prime }}(t)\sigma (t_{0})+\frac{\dot{E}(t)}{E^{2}(t)}\sigma (t)+{\dot{\varepsilon }}_{sh}(t)\right\}$	$E_{ce}(t)\frac{\dot{E}(t)}{E(t_{0})}\varepsilon_{ms}(t)$
Case 7	$\frac{E(t_{0})}{1+E(t_{0})J^{\prime }(t)}$	$-E_{ce}(t)\left\{\dot{J^{\prime }}(t)\sigma (t)+{\dot{\varepsilon }}_{sh}(t)\right\}$	-
Case 8	$\frac{E(t_{0})}{1+E(t_{0}){\dot{J^{\prime }}}_{\alpha }(t)}$	$-E_{ce}(t)\left\{{\dot{\varepsilon }}_{\alpha }(t)+{\dot{\varepsilon }}_{sh}(t)\right\}$	-
Case 9	$\frac{E(t_{0})}{1+\chi (t)E(t_{0})J^{\prime }(t)}$	$-E_{ce}(t)\left\{\dot{J^{\prime }}(t)\sigma (t_{0})+{\dot{\varepsilon }}_{sh}(t)\right\}$	-

**Table 5:** Empirical equations for the two test series A and B.

Test Series	Parameters									
	$a_{1}$	$b_{1}$	$a_{2}$	$b_{2}$	$a_{3}$	$b_{3}$	$t_{0}$ (Days)	$E_{c}(t=t_{0})$ (MPa)	$E_{c}(t=28)$ (MPa)	${f^{\prime }}_{c}$ (MPa)	
A	9.5	1.7	51	1.45	4	0.85	10	29,400	32,800	30	
B	11.4	5.1	48.4	0.73	2.6	0.9	7	22,700	25,300	28	
$J^{\prime }(t,t_{0})=\frac{{\left(t-t_{0}\right)}^{0.6}}{a_{1}+{\left(t-t_{0}\right)}^{0.6}}\frac{b_{1}}{E_{c}(t=t_{0})}$, $\varepsilon_{sh}(t,t_{0})=\frac{\left(t-t_{0}\right)}{a_{2}+b_{2}(t-t_{0})}\varepsilon_{shu}$, $E_{c}(t)=\sqrt{\frac{t}{a_{3}+b_{3}t}}E_{c}(t=28\;days)$										
